# Empowering community pharmacists: expert consensus guidance for the effective management of peripheral neuropathy with neurotropic B vitamins

**DOI:** 10.1186/s40780-026-00600-3

**Published:** 2026-06-20

**Authors:** Yolanda R. Robles, Lusy Noviani, Kitiyot Yotsombut, Grace Chew, Kenny James Merin, Navin Kumar Loganadan, Camilla Bennett

**Affiliations:** 1 College of Pharmacy, University of the Philippines, Ermita, Manila 1000 Philippines; 2 Department of Pharmacy, Atma Jaya Catholic University of Indonesia, Jakarta Barat, 12930 Indonesia; 3 Department of Pharmacy Practice, Chulalongkorn University, Bangkok, 10330 Thailand; 4 Independent Pharmacist Researcher, Singapore, Singapore; 5 College of Health Sciences, Lyceum of the Philippines, Davao, Davao City, 8000 Philippines; 6 Hospital Putrajaya, Putrajaya, 62250 Malaysia; 7 NSW Health, NSW, 2024 Australia

**Keywords:** Peripheral neuropathy, Neurotropic B vitamins, B Vitamins, Patient management, Community pharmacists, Delphi method

## Abstract

**Introduction:**

Peripheral neuropathy (PN) is a prevalent condition in the Asia-Pacific region with a significant proportion of undiagnosed cases. Despite heterogeneous pharmacy practices across the region, clinical and operational challenges remain consistent. Community pharmacists are well-positioned to overcome these challenges but lack specific guidance for managing PN in the pharmacy setting.

**Aim:**

This study aimed to develop expert consensus recommendations for community pharmacists on the early screening, assessment and management of PN.

**Method:**

A modified two-round Delphi process was adopted, involving a panel of seven pharmacists from across the region. The process included a targeted literature review, yielding a total of 41 articles, two rounds of anonymous survey, and a virtual expert round table discussion (ERTD) with the panel of pharmacists to achieve consensus.

**Results:**

The Delphi process yielded six consensus statements, covering the burden of PN, role of community pharmacists, early screening of patients at risk of PN, assessment and evaluation of PN patients, and guidance on management approaches of PN with neurotropic B vitamins.

**Conclusion:**

These consensus statements offer practical guidance for community pharmacists to improve the early detection and management of PN in the Asia-Pacific (APAC) region, potentially enhancing patient outcomes and quality of life.

**Supplementary Information:**

The online version contains supplementary material available at 10.1186/s40780-026-00600-3.

## Background

Peripheral neuropathy (PN) is a widespread and often debilitating condition arising with a global prevalence of up to 7% [1, 2]. This burden is particularly substantial and growing in the APAC region, largely driven by the high prevalence of diabetes with diabetic peripheral neuropathy (DPN) affecting approximately half of all patients with diabetes [3]. Studies across the APAC region report significant prevalence rates, reaching up to 21% in Australia [4], 58% in the Philippines [5–7], 54% in Malaysia [5], 58% in Indonesia [5] and 28% in Singapore [8]. A clear understanding of the regional burden of PN is concealed by a variety of clinical and patient-related factors. For healthcare providers, diagnosis can often be hindered by a tendency to focus solely on underlying health conditions, such as diabetes, rather than the neuropathy itself. Additionally, factors like the underestimation of neuropathy’s severity and the time constraints of a typical clinical appointment can pose significant challenges. Patients also face barriers which contribute to delays in diagnosis, including difficulty in articulating the specific and often progressive symptoms. Furthermore, there is a common tendency for individuals to ignore early-stage symptoms or misattribute them to other causes, further delaying a formal diagnosis. This contributes to a significant undiagnosed population, with one recent study suggesting that over 80% of individuals with PN have not received a formal diagnosis [9].

The pathophysiology of PN involves damage to peripheral nerve fibres, stemming from a multitude of causes including metabolic conditions like diabetes, mechanical nerve injury, excessive alcohol consumption, and nutritional deficiencies, particularly of B vitamins [10–12]. This damage disrupts nerve signalling, and patients commonly experience numbness, tingling, burning sensations, and nerve pain, typically in the hands and feet [13–15]. The impact of PN extends far beyond these immediate symptoms, often leading to severe morbidities that significantly diminishes a patient’s quality of life [16]. This includes muscle weakness, impaired balance leading to falls and foot ulcers that can result in amputation. These morbidities impact patients’ quality of life [17], leading to depression and anxiety [18], increased risk of cardiovascular complications, impacted sleep [19–21] and mortality. In fact, an estimated 57% of PN patients report substantial interference on sleep [22, 23].

Current management strategies for PN primarily involve prescription medications such as anticonvulsants, certain antidepressants, opioids, and local anaesthetics [14, 24–27]. While these drugs can provide rapid relief for some individuals, their utility is often limited. These medications are typically reserved for neuropathic pain, which represents a later stage of nerve damage that has progressed from early mild or moderate PN. Their use is also associated with a challenging side-effect profile, including dizziness, fatigue, and weight gain, which can make them difficult for patients to tolerate long-term and results in only partial pain control [28].

Beyond these pharmacological concerns, a consistent long-term management approach for PN is crucial. Pharmacists are pivotal in this management continuum, offering valuable guidance and sustained support. They have the advantage of early patient engagement, often preceding primary physician consultations. This positions them uniquely to screen for potential at-risk patients and recommend appropriate treatment options that present an excellent risk-benefit profile and are readily available over-the-counter (OTC). The availability of these treatments in pharmacies ensures prompt accessibility and allows pharmacists to provide timely, accessible treatment advice at the onset of symptoms, enhancing patient outcomes effectively.

Therapeutic dose of B vitamins complex has been widely used in clinical practices across APAC for selective patient groups [29, 30], with evidence suggesting their role in nerve health (e.g. nerve repair, energy metabolism, myelin sheath regeneration) [31–47]. However, while other non-pharmacological therapies like physical and psychosocial support are recommended, there remains a gap concerning the limited inclusion of a complex of neurotropic B vitamins (B1, B6, and B12) [13, 14, 27, 48].

Amidst these challenges, community pharmacists are increasingly recognized as crucial healthcare providers integral to primary care, especially in the management of certain chronic conditions. Their role in delivering public health services, including health screening, minor ailment management, OTC medication dispensing, medication review or optimization and patient education [49–57], positions them as a crucial first point of contact for many patients, especially in the APAC region [11, 58, 59]. By leveraging their expertise, pharmacists can help address the significant undiagnosed burden of PN through early screening, recommend early treatment options with OTC medication, and appropriate referral, preventing disease progression and severe complications [6, 60].

However, the effective integration of pharmacists into PN management is limited by heterogeneous pharmacy practices across the region and a lack of clear, evidence-based guidance specifically in the community pharmacy setting and for PN management with neurotropic B vitamins. Therefore, there is a pressing need to develop clear, practical, and evidence-based guidance.

To address this gap, the aim of this expert consensus guidance is to provide community pharmacists with a comprehensive set of recommendations for the early screening, assessment, and management of PN in the community pharmacy setting with neurotropic B vitamins. By empowering pharmacists with the necessary knowledge and tools, we aim to standardize care, improve the early detection of PN, and ultimately enhance the quality of life for patients.

## Methods

### Overview of study design

**Fig. 1:**
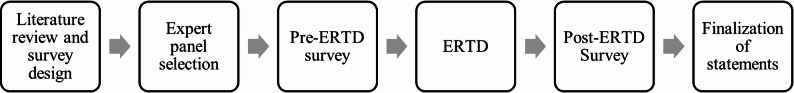
Workflow: two-round Delphi Method approach

The study design involved a targeted literature review and a modified two-round Delphi method, as outlined in Fig. 1.

### Selection of expert panel members

While there is no universally fixed minimum panel size, some have recommended seven per stakeholder group [61]. In addition, given feasibility constraints in this area – there are relatively few pharmacists across APAC with substantial experience, particularly with PN – the use of a seven-member panel was pragmatic. Hence, a panel of seven expert pharmacists was assembled to provide a breadth of international perspectives, with members from Australia, Indonesia, Malaysia, Philippines, Singapore and Thailand (Table A1). Recruitment used a free-finding approach: candidates were identified from publicly available sources (national pharmacy association directories, hospital and university websites) and via professional networks and peer referral to maximise variation in country and practice setting. Eligible experts were invited and enrolled upon providing written informed consent. The selection of these experts was guided by specific inclusion criteria, requiring pharmacist currently/previously based in the retail or hospital pharmacy setting for diversity of practice, experienced with managing patients with PN, have seen more than 30 PN or nerve damage patients in the past 3 months or more than 10 years of experience in their current specialty, good familiarity with the current landscape and management of PN or nerve damage, based in Australia, Indonesia, Malaysia, Philippines, Singapore, or Thailand. These selection criteria ensured coverage of domains relevant to the topic. We mitigated the small panel size by anchoring statements in a targeted literature review (41 articles appraised with Grading of Recommendations Assessment, Development and Evaluation (GRADE)), using two Delphi rounds plus an expert round table, and focusing the guidance on cross‑cutting pharmacist roles that are adaptable to local regulations. Experts were excluded if they declined to provide informed consent or do not qualify under the inclusion criteria.

### Targeted literature review

A targeted literature review using thematic analysis was conducted to establish an evidence base on PN management in community pharmacies across the APAC region. Given the objective to anchor a Delphi process rather than produce a comprehensive systematic review, we used PubMed as the primary source due to its broad biomedical coverage, Medical Subject Heading (MeSH) indexing and strong capture of APAC publications. Grey literature sources were also used to supplement the findings. Inclusion criteria were English-language, human studies or guidance relevant to adults with PN or at risk of PN in APAC, covering epidemiology/burden, screening/assessment tools, pharmacist roles (screening, counselling, referral), and PN management (pharmacological and non-pharmacological, including B vitamins). Eligible study types included clinical guidelines/consensus statements, randomized and non-randomized clinical trials, observational studies, systematic reviews, and qualitative studies informing pharmacist practice. Exclusion criteria were paediatric-only populations; case reports; editorials or letters without primary data; conference abstracts without full text; studies focused exclusively on inpatient/surgical settings or highly specialized diagnostics not transferable to community pharmacy; non-English publications; and inaccessible full texts. The quality of the evidence from these articles was systematically appraised via the GRADE methodology. Two independent reviewers (YR, CB) screened and graded the literature, with a third reviewer (LN) resolving any conflicts. The GRADE approach allows for a comprehensive evaluation of various study types, providing a necessary and structured evidence summary to inform expert clinical consensus and subsequent recommendations.

### Surveys and ERTD

Following the literature review, a set of initial statements was first developed. In the first round of anonymous surveys, the expert panel was asked to rate their agreement with a set of statements on a 10-point Likert scale. Each expert had one vote of equal weight. Following established Delphi practices in the field, consensus for each statement was achieved if at least 80% of the experts rated the level of agreement as 8 or above on a 10-point Likert scale [59, 62]. Statements not meeting this criterion were identified and prioritised for the ERTD. During the ERTD, all statements were debated and revised based on the panel’s qualitative input, with a stronger focus on the statements that did not achieve consensus in the first round of survey. After the ERTD, a second round of survey was then administered to rate the revised statements. The ERTD was moderated by an independent moderator (AG) not involved in drafting nor rating of the statements to minimize bias. Differing opinions were discussed to allow all experts to articulate concerns; if disagreement persist, the statement would be categorized as ‘no consensus’.

## Results

### Targeted literature review

**Fig. 2:**
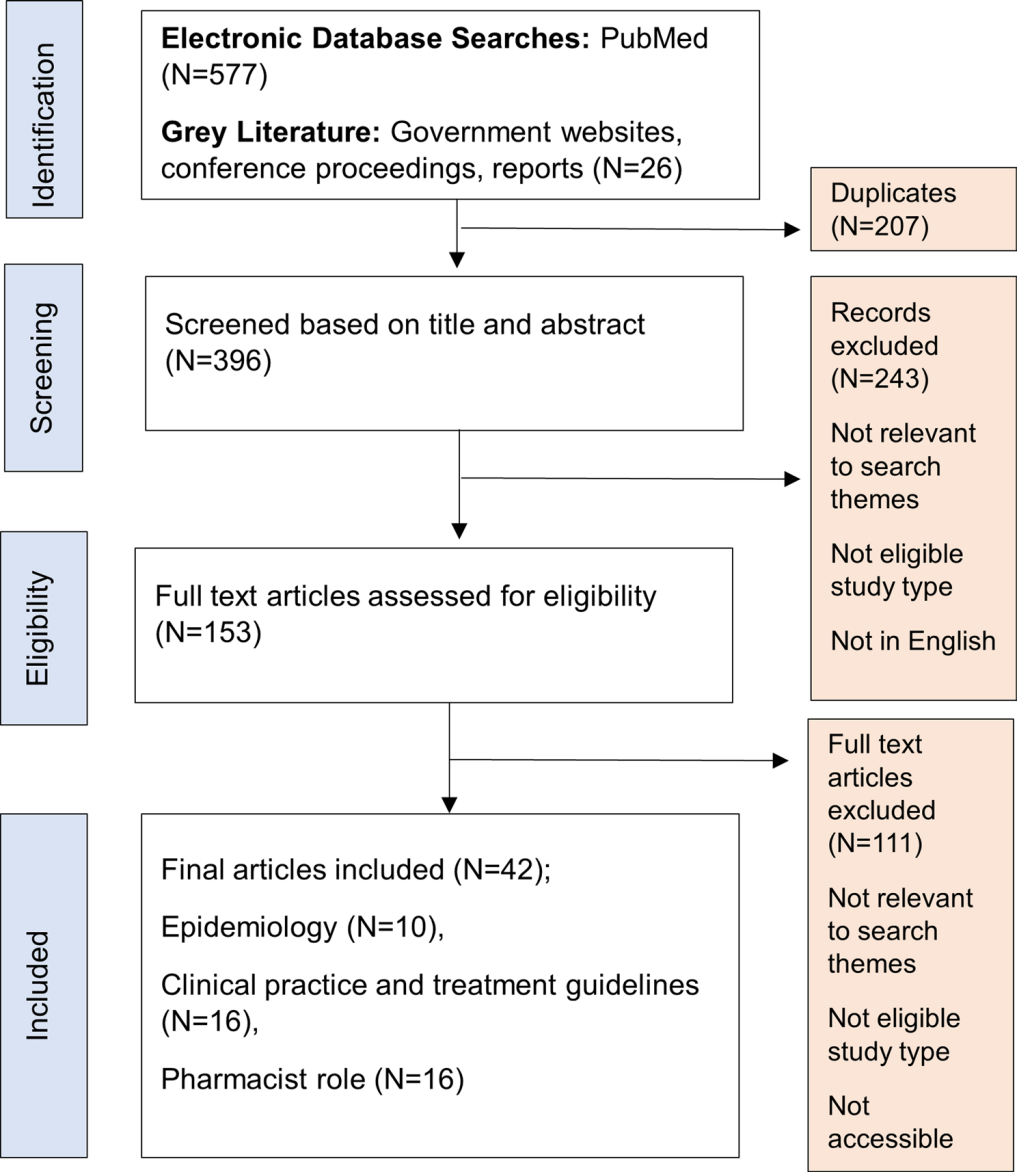
Study selection flow (PRISMA diagram)

The article identification and selection process are outlined in a Preferred Reporting Items for Systematic Reviews and Meta-Analyses (PRISMA) diagram (Fig. 2), which concluded with 41 articles being included. These publications served as the basis for the initial consensus statements.

A comprehensive search of the PubMed database, spanning from January 27, 2015, to January 27, 2025, initially identified 577 records (detailed search terms can be found in Table A2). Additionally, 26 records were identified from grey literature sources, including government websites, conference proceedings, and reports. After removing 207 duplicates, the remaining 396 records were screened by title and abstract against the above criteria. 243 records were excluded as they were either not relevant to the search themes as stated above, not the right study type, or not in English. Of the remaining 153 full text articles assessed for eligibility, 111 articles were excluded as they were either not relevant to the search themes as stated above, not the right study type, or not accessible. 42 final articles were included, of which 10 were related to epidemiology, 16 were related to clinical practice and treatment guidelines, and 16 were related to the role of pharmacists.

### Consensus statements

In Round 1 of the survey, 5 out of 6 statements met the consensus threshold of 80% of experts rating agreement as ≥ 8.0 out of a 10-point Likert scale, with mean scores ranging from 7.14 to 9.57. All statements were discussed and revised during the ERTD, with a priority to revise the one statement that did not meet the threshold, which was Statement 5. In Round 2, all six statements achieved consensus, with mean scores ranging from 9.0 to 9.9. The mean score for each statement in Round 1 and Round 2, as well as the changes made in each statement can be found in Table A3.

The final statements cover the topics of the role of community pharmacists in management, early screening tools, symptoms and treatment algorithm of PN. Additionally, Table 1 provides a summary of these statements, accompanied by the average level of agreement on a 10-point Likert scale. Further details on the final statements and their supporting literature were provided in Table A4.

**Table 1:** Summary of endorsed consensus statements

No.	Consensus statement	Level of agreement
1	Community pharmacists should play an active role in the management of PN, particularly given its high prevalence among diabetic patients, ranging from 27% to 58%, with approximately 80% of PN cases remaining undiagnosed.	9.7
2	The role of pharmacists extends to - 1. The proactive early screening and management of mild to moderate PN 2. Recommending appropriate OTC and dispensing prescription medication 3. Providing health counselling and follow-up activities 4. Advising on when to refer patients to a general practitioner for further evaluation.	9.6
3	To identify patients at risk of PN, pharmacists can utilize the mnemonic MEDIC as shown in Table 2.	9.0
4	Common clinical symptoms for PN include tingling, numbness and nerve pain. Pharmacists can employ initial evaluation tools, such as clinical and lifestyle history, screening questionnaires (such as A Simple SCreening Tool (ACT), Douleur Neuropathique 4 (DN4), Neuropathic Pain Questionnaire (NPQ)) and sensory examinations (if local regulations permit), to assess patients with PN in the pharmacy setting.	9.3
5	Management of PN patients by pharmacists is recommended to follow the treatment algorithm in Fig. 3. Pharmacists may recommend pharmacological approaches, including a complex of therapeutic doses of B vitamins (B1, B6, B12), low-dose pain relievers, and topical treatments for managing early PN.	9.0
6	Pharmacists should provide clear counselling to patients regarding the use of pharmacological options such as a complex of therapeutic doses of B vitamins (B1, B6, B12), including guidance on appropriate dosing, duration of therapy and potential side effects. This ensures patient safety while contributing to improved overall care.	9.9

#### Disease burden of PN in the APAC region

##### Statement 1

Community pharmacists should play an active role in the management of PN, particularly given its high prevalence among diabetic patients, ranging from 27% to 58%, with approximately 80% of PN cases remaining undiagnosed.

The expert panel highlighted the crucial role of community pharmacists in addressing the significant public health concern of PN, particularly within the diabetic population. They noted that pharmacists’ engagement can not only ease the workload of physicians but also facilitate early detection and timely intervention for PN. This involvement aids in slowing disease progression, managing symptoms, and enhancing patients’ quality of life.

#### Role of community pharmacists in detecting and managing PN

##### Statement 2

The role of pharmacists extends to -

1. The proactive early screening and management of mild to moderate PN.
2. Recommending appropriate OTC and dispensing prescription medication.
3. Providing health counselling and follow-up activities.
4. Advising on when to refer patients to a general practitioner for further evaluation.

The expert panel emphasized the crucial integration of pharmacists within the broader healthcare ecosystem. Pharmacists should not only perform proactive screening, OTC recommendations, and medication dispensing but also play a key role in identifying potential drug-disease or drug-drug interactions, managing medication therapy, providing health counselling, and referring patients to physicians when necessary. The expert panel noted that this expanded scope is particularly vital for early intervention of PN, where pharmacists not only initiate proactive PN screening in high-risk population, and facilitate ongoing PN symptom monitoring, but also inter-professional communication with other healthcare providers. In Australia, pharmacist-led programs like Home Medicines Review (HMRs) [63] and MedsCheck [64] exist, which highlights potential for the integration of PN services into such programs in other markets. Furthermore, the integration of digital health tools, such as patient monitoring devices and specialized apps, empowers pharmacists to track patient symptoms and therapy outcomes with greater precision.

Crucially, a structured framework must be in place to define when a patient’s PN condition requires medical evaluation beyond what a pharmacist can provide. Establishing clear referral criteria is essential for efficient patient management and ensures that pharmacists are equipped to promptly direct PN individuals with worsening symptoms such as acutely progressive severe pain, motor weakness, or functionally limiting weakness to a physician (e.g. a general practitioner) or specialist (e.g. a neurologist) for further assessment [65].

#### Identification of PN in the community pharmacy setting: screening and assessment tools

##### Statement 3

To identify patients at risk of PN, pharmacists can utilize the mnemonic MEDIC as shown in Table 2.

**Table 2:** MEDIC mnemonic for identifying patients at high risk of PN

Mnemonic	Identifying patients at high risk of PN
M	**M**edication (e.g. Metformin, Isoniazid, Proton pump inhibitors)
E	**E**lderly (with increasing age)
D	**D**iabetes and diet
I	**I**nfection (Tuberculosis, herpes zoster)
C	**C**hronic kidney disease on dialysis

The expert panel suggested a simple but effective way for pharmacists to identify common patient groups at risk of PN through a mnemonic, representing key patients at risk of PN. MEDIC, developed during the ERTD, is a practical framework for community pharmacists to guide pharmacists in recognizing key risk factors during routine patient interactions. It is important to emphasize that the MEDIC is a framework allowing pharmacists to perform risk stratification and appropriate intervention, from OTC recommendation to referral, and not to diagnose patients with PN. Identifying patients at risk of PN or red flags, such as rapidly progressing symptoms or severe pain, is essential to ensure proper follow up and referral of patients for further evaluation and timely treatment.

The MEDIC mnemonic encompasses several well-established risk factors for PN:

- **M**edication: certain medications such as metformin, isoniazid and proton-pump inhibitors, have been associated with an increased risk of PN [66–68]. Metformin use is associated with an 84% increased risk of DPN when compared with patients not using metformin [67], while it has been estimated that isoniazid is commonly associated with PN development in as many as 10% of the patients [66].
- **E**lderly: the risk of PN increases with age, with older adults being at greater risk of PN [69]. In a review by Head (2006), the incidence of PN increased from 8.1% amongst individuals aged 40–49 year to 34.7% in individuals over age of 80 [70], likely due to presence of comorbidities including diabetes in older adults.
- **D**iabetes and diet: as a leading cause of PN, a significant proportion of diabetic patients develop neuropathic complications [71]. In the APAC countries, PN occurs in approximately 50% of the diabetes population, ranging from 33% in Singapore to 58% in Philippines [5]. Additionally, patients with special diets such as vegetarian, may risk nutritional deficiency from vitamin B12 and omega-3 fatty acids, resulting in higher risk for PN [72].
- **I**nfection: certain infections such as tuberculosis, herpes zoster virus, and human immunodeficiency virus (HIV) can contribute to the development of PN. The prevalence of infectious diseases varies geographically, influencing local relevance of this risk factor [66, 73], with prevalence of neuropathy among HIV-infected patients estimated to be 28% in Thailand [74].
- **C**hronic kidney disease (CKD): patients with CKD on dialysis are associated with an increased risk of PN [75, 76], with over 63% of CKD patients reported having PN [77].

Pharmacists can ascertain patient risk factors through patient interactions, examining medical and medication histories, and considering the demographic context of each patient (as outlined in consensus statement 2 on the pharmacists’ role). Acknowledging the diverse practice settings across the region, the expert panel emphasized that the mnemonic can be adapted to align with local cultures and languages in the region, empowering pharmacists to optimize patient care practices within their specific contexts. Additionally, while the established risk factors for PN are well-documented, this list is not exhaustive. Non-listed causes contributing to PN including chemotherapy-induced neuropathy [78], cancer-related conditions and pharmacists encountering patients receiving these therapies should refer them to their treating specialists. As this is a suggested framework that emerged from this study, prospective validation is needed to ensure real-world feasibility and practice for PN patients in the community pharmacy setting.

##### Statement 4

Common clinical symptoms for PN include tingling, numbness and nerve pain. Pharmacists can employ initial evaluation tools, such as clinical and lifestyle history, screening questionnaires (such as A Simple SCreening Tool (ACT), Douleur Neuropathique 4 (DN4), Neuropathic Pain Questionnaire (NPQ)) and sensory examinations (if local regulations permit), to assess patients with PN in the pharmacy setting.

**Table 3:** Key differences between nerve pain and muscle pain

	Nerve pain (PN) [10, 12]	Muscle pain [79]
Signs and symptoms	Burning pain	Aching
	Shooting pain	Spasm and cramps
	Prickling sensation	Stiffness
Type of pain	Sharp	Dull
Onset of pain	Sudden	Constant
Trigger of pain	Often aggravated while still	Often worse while moving
Body part	Starts from extremities and progresses up towards central nervous system	Legs, arms, shoulders

The expert panel identified key differences between nerve pain and muscle pain that would help community pharmacists when assessing PN patients (Table 3). They recommended pharmacists to adopt standardized evaluation tools such as ACT (Figure A1, Figure A2) [9] to distinguish between mild and severe symptoms, as well as DN4 [80] and NPQ [81]. These tools are non-invasive, quick to administer and widely relevant for pharmacists in the region. Furthermore, documenting all findings, including clinical and lifestyle history, using standardized forms facilitates data comparison and aggregation.

However, the expert panel acknowledged the variability in training practices and regulatory allowance across countries. In certain countries, including Thailand, physical sensory examinations such as monofilament or pinprick testing are legally restricted to physicians [82]. Therefore, pharmacists in these settings should focus on non-invasive, interview-based methods when evaluating potential PN patients.

#### Management of PN: Recommendations for community pharmacists

##### Statement 5

**Fig. 3:**
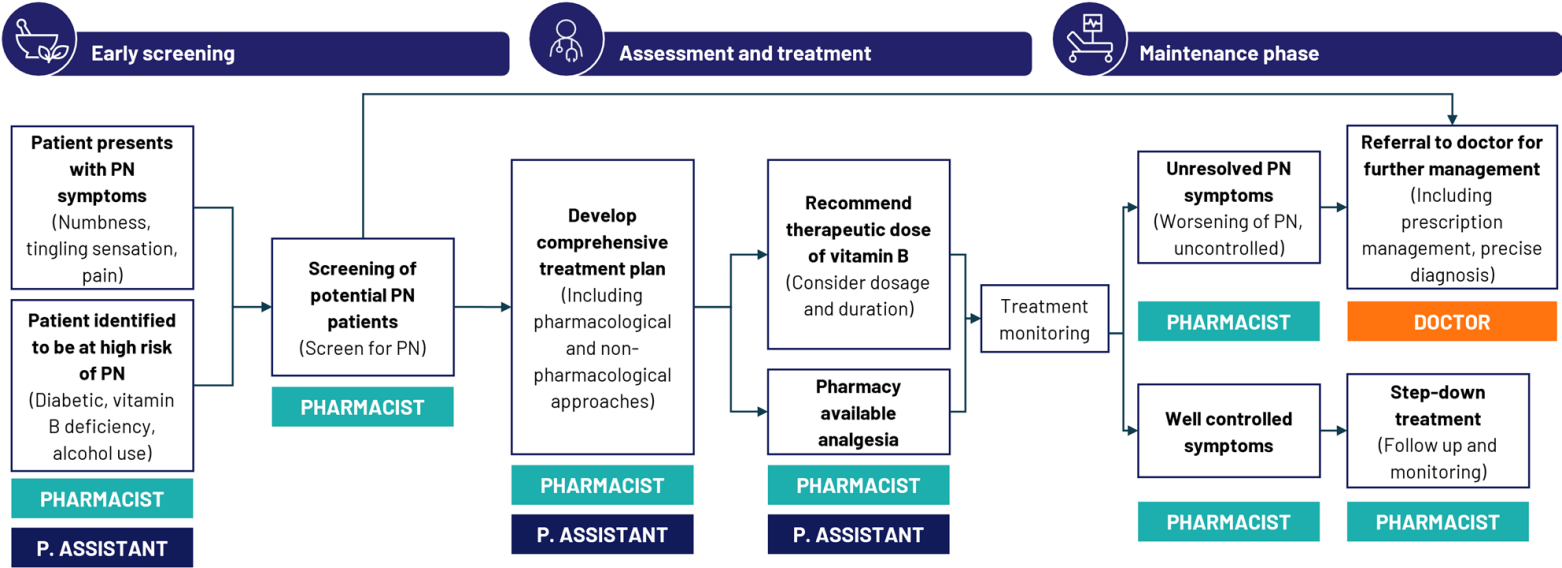
Treatment algorithm for management of PN patients

Management of PN patients by pharmacists is recommended to follow the treatment algorithm in Fig. 3. Pharmacists may recommend pharmacological approaches, including a complex of therapeutic doses of B vitamins (B1, B6, B12), low-dose pain relievers, and topical treatments for managing early PN.

The expert panel presents a proposed treatment algorithm (Fig. 3) designed to guide pharmacists in managing potential PN patients. This algorithm is structured into three distinct phases: Early Screening, Assessment and Treatment, and Maintenance.

Upon presentation at a community pharmacy, patients identified as high-risk (as listed in Statement 3) by the pharmacist or pharmacy assistant or those exhibiting PN symptoms (as previously outlined in Statement 4) should undergo initial screening by the pharmacist using the appropriate screening tools. This early screening is crucial for identifying early cases of PN and providing early management advice to patients. Following screening, patient management proceeds based on the severity of suspected PN. For patients identified as at risk or in the early stages of PN with mild symptoms, pharmacists can recommend appropriate OTC medications as part of a proactive management strategy. On the other hand, pharmacists should immediately refer patients with more severe symptoms to a physician for precise diagnosis and comprehensive medical evaluation.

For patients managed within the pharmacy, pharmacists with operational support from the pharmacy assistants should formulate a holistic treatment plan encompassing both pharmacological and non-pharmacological strategies. This includes patients already diagnosed with PN and prescribed with a low dose of B vitamins but are still experiencing unresolved symptoms. Pharmacological interventions, while ideally tailored to the underlying etiology, generally could include a complex of therapeutic dose of B vitamins (B1, B6, B12) or topical OTC products containing capsaicin (0.075%) [83] or lidocaine (5%) [84]. Pharmacists may consider recommending a complex of therapeutic-dose B vitamins as adjunctive therapy alongside prescribed agents such as pregabalin or gabapentin where clinically appropriate, with the aim of achieving additive or synergistic benefits [85, 86]. Table 4 below outlines the recommended therapeutic oral dose of vitamin B1, B6 and B12 for neuropathy based on available literature. The onset of efficacy in relieving neuropathy symptoms has been reported in as early as 2 weeks, with progressive improvement throughout the treatment duration of 3 months without plateau [33]. Patients undergoing treatment should be monitored for three to six months before undergoing re-evaluation by the pharmacist. Should symptoms of PN persist or worsen, the pharmacist should promptly refer patients to the doctor for further management.

**Table 4:** Recommended oral dose of vitamin B1, B6 and vitamin B12 for different populations

	Daily Recommended Dietary Allowance (RDA), for healthy population	Therapeutic Oral Dose, for neuropathy (nerve damage)
Vitamin B1	1.2 mg [87–89]	100–400 mg [36, 42, 47, 90]
Vitamin B6	1.3 mg [88, 89, 91]	50–600 mg [39, 92–95]
Vitamin B12	2.4–4.0 mcg [88, 89, 96]	200–5000 mcg [32, 33, 37, 39]

The panel noted that a critical component of this integration is comprehensive pharmacist training, particularly regarding the appropriate dosage and duration of neurotropic B vitamins, where knowledge gaps currently exist within the region.

##### Statement 6

Pharmacists should provide clear counselling to patients regarding the use of pharmacological options such as a complex of therapeutic doses of B vitamins (B1, B6, B12), including guidance on appropriate dosing, duration of therapy and potential side effects. This ensures patient safety while contributing to improved overall care.

The expert panel acknowledged the role played by community pharmacists in patient education in PN. By providing clear and comprehensive counselling to patients experiencing symptoms of PN, especially when recommending pharmacological interventions, pharmacists can significantly mitigate risks associated with improper medication use.

Following this, precise guidance on dosing is essential. Pharmacists should detail the exact dosage and duration for B vitamin intake, considering individual patient factors such as their specific condition, medication history and comorbidities. In addition, pharmacists should thoroughly discuss potential side effects associated with the various pharmacological approaches (such as drowsiness and gastric discomfort) and flag any relevant drug interactions. Vitamins B1 and B12 generally exhibit favourable safety profiles with no recognized upper intake limits due to efficient bodily excretion of any excess consumed, and are hence safe for long-term use [97, 98]. However, prolonged usage (e.g. > 6 months) of high doses (e.g. > 600 mg) of vitamin B6 has been observed to paradoxically induce neurological symptoms. To ensure safe usage of vitamin B6, the following tolerable (maximum) daily dosage and associated duration of vitamin B6 intake identified by Schellack et al. [99] and summarized in Table 5 can be referenced.

**Table 5:** Tolerable (Maximum) daily dosage and duration of vitamin B6

Tolerable (Maximum) Dosage and Duration According to Literature [99]	
**Daily dose of Vitamin B6**	**Duration of Vitamin B6 Intake**
	**Patients with PN**
50 mg	Long term use possible
100 mg	Up to 5 years
200 mg	Up to 200 days
Up to 600 mg	Up to 18 weeks

Patients should be made aware of the warnings on the label and instructed on how to identify potential adverse reactions and what steps to take if side effects occur, including making follow-up visits to the pharmacy where they can be referred to a general practitioner for further monitoring. The general practitioner may conduct neurological examinations and vitamin B6 tests to measure vitamin B6 levels, implementing a washout period to alleviate side effects if necessary [99]. Finally, practical information such as proper storage instructions for neurotropic B vitamins should be clearly communicated to patients to ensure optimal clinical effects.

## Discussion

As some of the most accessible healthcare professionals, community pharmacists are often the first point of contact for individuals seeking health advice or OTC remedies [100]. This accessibility creates a unique opportunity for pharmacists to identify people at risk for PN through routine interactions and targeted questioning. In countries such as Indonesia with a less favourable doctor-to-population ratio of 0.31 per 1,000 people [101], the role of pharmacists becomes even more critical in bridging the gap in primary healthcare access.

At the primary healthcare level, community pharmacists play role in managing PN by providing most appropriate recommendation to PN patients. This extends beyond traditional dispensing to include proactive screening, OTC recommendations, identifying drug interactions, medication therapy management (MTM), health counselling, and when necessary, referrals to physicians [54, 102]. These roles are also well defined in countries such as Australia [103] and Singapore [104], where pharmacists are guided by locally developed professional practice standards. Specifically, for management of mild to moderate PN, pharmacists can recommend non-pharmacological strategies that have demonstrated clear benefits, such as smoking cessation, moderating alcohol consumption, adopting a balanced diet, and engaging in regular physical activity. These non-pharmacological strategies [105] have proved particularly beneficial in patients with mild-moderate PN.

The expert panel underscores the vital role pharmacists play in the management of PN through the careful oversight of B vitamin therapeutics, often self-administered by patients in incorrect dosages or durations [106, 107]. Pharmacists provide critical patient education on correct medication practices, establishing structured frameworks for when a patient’s condition requires medical referral for further evaluation. This collaborative approach between pharmacists and healthcare providers ensures efficient patient management and outcome optimization. Expanding the pharmacist’s role to include active PN screening and management would position them—where permitted by local regulations—to dispense medications and provide counselling on dosage, duration, administration, and potential drug interactions (e.g., with tricyclic antidepressants), thereby supporting improved therapeutic outcomes [49–53]. Coordination between pharmacists and other healthcare providers is essential to maximise the effectiveness of PN management, creating a collaborative and patient-centred approach to care [50, 51]. This is especially critical when recommending a complex of therapeutic doses of neurotropic B vitamins as many patients may lack accurate information regarding appropriate dosage and duration. When counselling patients, pharmacists must clearly differentiate between a complex of therapeutic doses of neurotropic B vitamins, which is higher and specifically targets deficiencies or conditions like PN [106], and a general dietary supplement, which contains lower doses for nutritional support.

The proposal of a mnemonic tool, MEDIC, developed in this study, is anticipated to help community pharmacists assess patients for PN risk factors and red flags, rather than diagnosing the condition itself. By asking targeted questions about a patient’s symptoms, medical history, and lifestyle, pharmacists can easily determine if an urgent referral is needed. The tool is highly adaptable to different languages and cultures, and it reminds pharmacists to stay alert for a wide range of potential causes such as chronic alcoholism, chemotherapy, or nutritional deficiencies, ensuring comprehensive patient care across diverse practice settings.

Beyond pharmacological interventions, non-pharmacological strategies such as foot care, lifestyle modifications, and patient education are invaluable for managing mild to moderate PN. Pharmacists are well-positioned to reinforce these multimodal strategies and integrate them with pharmacological treatments [108]. However, while this proposed treatment algorithm is scientifically sound, its success in practice hinges on its integration into everyday pharmacy practice and how well it empowers pharmacists to manage potential PN patients prior to formal diagnosis. Investing in robust pharmacist education is paramount to translating this algorithm into effective clinical practice and ultimately improving patient outcomes.

Familiarity with standardized evaluation tools such as ACT (Figure A1, Figure A2) [9], DN4 [80] and NPQ [81] is essential for pharmacists to conduct accurate assessments of PN. Despite the availability of numerous screening questionnaires, many suffer from limitations like complexity and inconsistent scoring, which reduces their practical utility. The panel’s recommended tools overcome these barriers, paving the way for targeted interventions. Such assessments should be documented systematically, facilitating data comparison and improving insights into PN progression and treatment efficacy. Where regulations and training allow, sensory examinations can provide additional objective data, although in regions where these practices are restricted to physicians, pharmacists should rely on interview-based evaluations. Integrating these tools into routine pharmacy practice can significantly enhance the quality of care provided to patients with PN.

Looking ahead, emerging technologies such as Artificial Intelligence (AI)-powered decision support systems, mobile health applications, and digital symptom assessment platforms can empower pharmacists to better identify at-risk patients and offer personalized care. However, successfully using these tools in pharmacies requires comprehensive training. This investment in training is key to improving patient assessment and cementing the vital role of pharmacists in primary healthcare.

Although community pharmacists in the region generally provide counselling services alongside their traditional roles of managing care for minor ailments and dispensing medication, the public may not always be aware of such services [49–53, 57, 102, 109]. A survey conducted in the Philippines by Vreeland et al. revealed that while the general public was aware of community pharmacy practices like advising on minor ailments, their awareness of other services such as patient counselling and disease management was minimal, often leading them to seek information and advice from online resources instead [53]. Hence, there is a need to emphasize and promote the pharmacist’s role in patient counselling.

However, community pharmacists in the region face several systemic barriers that impede consistent patient education and counselling [102]. These challenges include significant time constraints, limited access to patients’ medical histories, and a lack of structured counselling tools. Implementing effective follow-up plans in the community pharmacy setting also presents difficulties, as patient engagement often declines once symptoms improve, and there is currently no formal system in place to monitor progress for non-prescription therapies. To address these challenges, pharmacy counselling can be enhanced by using checklists for core messages, training sessions on communication and product knowledge, and tools like logbooks and reminder systems to improve follow-up and treatment adherence. This guidance emphasizes integration of PN management into daily workflow through simplified screening frameworks to identify high-risk patient groups. Documenting and integrating patient information into accessible data systems for healthcare professionals will empower pharmacists, enhancing their role in long-term patient management.

Ultimately, well-informed patients are more likely to adhere to their prescribed regimens, leading directly to improved outcomes and overall health. By providing detailed guidance and support, pharmacists can solidify their position as an integral part of the patient’s healthcare journey, thereby elevating the quality of care and enhancing patient well-being within the community.

### Limitations

The study acknowledges several limitations impacting the implementation of the guidance. The proposed MEDIC framework, while simplified, requires further validation to ensure their efficacy in real-world community pharmacy settings. The limited panel size may affect generalizability to health systems with markedly different financing models, care pathways, or pharmacist scope of practice. Heterogeneous practice habits, referral pathway, pharmacist training practices, community pharmacy regulatory allowance and resource availability across the APAC region pose another limitation to effective implementation of a standardised guidance. While the current manuscript focus on community pharmacist’s roles in the management of PN, future iterations can be broadened to physicians (e.g. general practitioners, neurologists) and other allied healthcare professionals (e.g. dieticians) for cross-disciplinary collaborations on precise diagnosis, lifestyle management, and holistic treatment regimen to ensure effective implementation and further improve PN patient care.

## Conclusions

The management of PN represents a significant challenge, especially in the APAC region, where a substantial proportion of cases remain undiagnosed, resulting in delayed treatments.

This consensus statement publication synthesizes six key statements that address the burden of PN, clarify the role of pharmacists in PN management, and outline a treatment algorithm to provide practical, evidence-based guidance for pharmacists in the community pharmacy setting, including screening at-risk patients, conducting thorough assessments, and using a complex of therapeutic doses of B vitamins as adjunctive therapy to improve outcomes. Contextualizing for APAC’s heterogenous practice environments, the guidance is intended to complement existing tools in well-resource markets (E.g. Australia) while being actionable in settings with constrained access to diagnostics, specialist services and reimbursement. Implementing this guidance will require addressing systemic barriers such as time constraints and lack of training through comprehensive education across the six consensus items, supported by follow-up programs and data integration to sustain practice change. Future research should focus on evaluating the long-term effectiveness and cost-effectiveness of these guidance in real-world settings, exploring the impact of emerging technologies on PN management in the community pharmacy setting, identifying additional PN risk factors to further enhance these recommendations and expand broad application beyond the APAC region. Collectively, this pioneering initiative underscores the critical role of pharmacists as accessible healthcare providers in addressing a prevalent, yet often underdiagnosed condition in the APAC region, thereby fostering a collaborative approach to improve the management of PN within the community pharmacy setting.

## Supplementary Information

Below is the link to the electronic supplementary material.

Supplementary Material 1

## Data Availability

The original contributions presented in this study are included in the article/ supplementary material. Further inquiries can be directed to the corresponding author(s).

## Abbreviations

ACT: A simple sCreening Tool
AI: Artificial Intelligence
APAC: Asia-Pacific
CKD: Chronic Kidney Disease
DN4: Douleur Neuropathique 4
DPN: Diabetic Peripheral Neuropathy
ERTD: Expert Round-table Discussion
GRADE: Grading of Recommendations Assessment, Development and Evaluation
HIV: Human Immunodeficiency Virus
HMR: Home Medicines Review
MEDIC: Medication; Elderly; Diabetes and diet; Infection; Chronic kidney disease
MeSH: Medical Subject Headings
MTM: Medication Therapy Management
NPQ: Neuropathic Pain Questionnaire
OTC: Over-the-counter
PN: Peripheral Neuropathy
PRISMA: Preferred Reporting Items for Systematic Reviews and Meta-Analyses
RDA: Recommended Dietary Allowance
