# Supplementary material for: Empowering community pharmacists: expert consensus guidance for the effective management of peripheral neuropathy with neurotropic B vitamins
Source: J Pharm Health Care Sci. 2026 Jun 20;12:63. doi: 10.1186/s40780-026-00600-3 (PMC13366861; doi:10.1186/s40780-026-00600-3)
Supplement: Supplementary file 1 — Supplementary Material 1 [file 40780_2026_600_MOESM1_ESM.docx]

**Appendix A**

*Appendix A.1*

**Table A1.** Expert panel profiles

| **Name** | **Qualification** | **Affiliation** | **Years of experience** |
| --- | --- | --- | --- |
| Dr Yolanda R. Robles | 1. Professor, College of Pharmacy   President | 1. Philippines College of Pharmacy, University of the Philippines 2. Federation of Asian Pharmaceuticals Association (FAPA) | 40 years |
| Dr Kenny James P. Merin | Professor III, Pharmacy Program, College of Health Sciences | Lyceum of the Philippines University - Davao | 15 years |
| Dr. Apt. Lusy Noviani | Vice Secretary | Indonesian Pharmacist Association (IAI) | 33 years |
| Dr Navin Kumar Loganadan | Head of Ambulatory Pharmacy Services | Hospital Putrajaya  Malaysia | 21 years |
| Ms Camilla Bennett | Director of Pharmacy | War Memorial Hospital | 15 years |
| Dr Kitiyot Yotsombut | Assistant Professor | Department of Pharmacy Practice, Chulalongkorn University | 18 years |
| Ms Grace Chew | Principal Pharmacist | Independent Researcher | 14 years |

**Table A2.** Search strategy

| **Topic** | **Subtopic** | **Keyword cluster 1** | **Keyword cluster 2** |
| --- | --- | --- | --- |
| Epidemiology | Burden of disease (prevalence, incidence, morbidity) | Peripheral neuropathy | Epidemiology OR Prevalence OR Incidence OR Burden OR Morbidity |
| Treatment | Treatment guidelines and algorithm |  | Treatment guidelines OR Treatment algorithm |
|  | Vitamin B place in therapy |  | Place in therapy OR Dosage recommendation OR Duration recommendation |
|  | Current unmet need and treatment expectations |  | Unmet need OR Treatment expectations |
|  | PN screening and referral |  | Screening OR Referral pathway |
| Pharmacist role | Role of pharmacist | Peripheral neuropathy AND Pharmacist | Pharmacist role OR patient management OR patient diagnosis |
|  | Pharmacist awareness and perception on conducting early screenings for PN |  | (Perception OR awareness OR attitudes OR beliefs) AND (early screening OR early management OR referral) |

**Table A3.** Survey ratings and changes in statements between Round 1 and Round 2

| **No.** | **Round 1 statement** | **Round 1 survey ratings (Mean)** | **Changes in statement** | **Round 2 statement** | **Round 2 survey ratings (Mean)** |
| --- | --- | --- | --- | --- | --- |
| 1 | Pharmacists should actively participate in the management of Peripheral Neuropathy (PN), recognizing that the prevalence among diabetic patients ranges from 27% to 58%, with approximately 80% of PN cases remaining undiagnosed. | 8.71 | 1. Specified community pharmacists 2. Highlighted that prevalence among diabetic patients is particularly high | Community pharmacists should play an active role in the management of PN, particularly given its high prevalence among diabetic patients, ranging from 27% to 58%, with approximately 80% of PN cases remaining undiagnosed. | 9.7 |
| 2 | The role of pharmacists extends to the proactive early screening and management of mild to moderate PN, supporting patients through Over-the-Counter (OTC) medication recommendation and prescription medication dispensing, health counseling, and guidance on when to refer patients to a general practitioner or specialist for further evaluation. | 8.71 | 1. Presented in a point structure 2. Added “follow-up activities” (Point 3) | The role of pharmacists extends to -   1. The proactive early screening and management of mild to moderate PN 2. Recommending appropriate OTC and dispensing prescription medication 3. Providing health counselling and follow-up activities 4. Advising on when to refer patients to a general practitioner for further evaluation. | 9.6 |
| 3 | To identify patients at risk of PN, pharmacists can utilize the mnemonic CONDITION—Check for Organ dysfunction, Nutritional deficiency, Drug-Induced neuropathy, Toxins, Infections, and Other Neuropathy​. | 8.43 | 1. Modified mnemonic from CONDITION to MEDIC based on expert opinion of groups at high risk of PN | To identify patients at risk of PN, pharmacists can utilize the mnemonic MEDIC as shown in Table 2. | 9.0 |
| 4 | Common clinical symptoms for PN include tingling, numbness, nerve pain. Pharmacists can employ initial evaluation tools, such as clinical history, screening questionnaires and sensory examinations, to assess patients with neuropathic pain in the pharmacy setting. | 8.43 | 1. Provided examples of specific screening tools 2. Added a caveat that sensory examinations are only to be performed if local regulations permit | Common clinical symptoms for PN include tingling, numbness and nerve pain. Pharmacists can employ initial evaluation tools, such as clinical and lifestyle history, screening questionnaires (such as A Simple SCreening Tool (ACT), Douleur Neuropathique 4 (DN4), Neuropathic Pain Questionnaire (NPQ)) and sensory examinations (if local regulations permit), to assess patients with PN in the pharmacy setting. | 9.3 |
| 5 | Management of PN patients by pharmacists is recommended to follow the following treatment algorithm (Figure 6). Pharmacists may recommend pharmacological approaches, including therapeutic doses of Neurotropic B vitamins (B1, B6, B12), low-dose pain relievers, and topical treatments for managing early PN. | 7.14 | 1. Removed diagnosis aspects from treatment algorithm as that pertains more to clinicians, focusing on pharmacists’ role instead | Management of PN patients by pharmacists is recommended to follow the treatment algorithm in Figure 3. Pharmacists may recommend pharmacological approaches, including a complex of therapeutic doses of B vitamins (B1, B6, B12), low-dose pain relievers, and topical treatments for managing early PN. | 9.0 |
| 6 | Pharmacists should provide clear counseling to patients regarding the usage of pharmacological options such as therapeutic-dose B vitamins, including dosing information, duration and potential side effects, while ensuring safety of patient and improving patient care. | 9.57 | 1. Specified the type of vitamin B when referring to therapeutic dose B vitamins 2. Minor changes in wording and sentence structure | Pharmacists should provide clear counselling to patients regarding the use of pharmacological options such as a complex of therapeutic doses of B vitamins (B1, B6, B12), including guidance on appropriate dosing, duration of therapy and potential side effects. This ensures patient safety while contributing to improved overall care. | 9.9 |

**Table A4.** Consolidated consensus statements and references

| **No.** | **Consensus statement** | **References** |
| --- | --- | --- |
| 1 | Community pharmacists should play an active role in the management of PN, particularly given its high prevalence among diabetic patients, ranging from 27% to 58%, with approximately 80% of PN cases remaining undiagnosed. | [1–7] |
| 2 | The role of pharmacists extends to -   1. The proactive early screening and management of mild to moderate PN 2. Recommending appropriate OTC and dispensing prescription medication 3. Providing health counselling and follow-up activities 4. Advising on when to refer patients to a general practitioner for further evaluation. | [2,6,8–20] |
| 3 | To identify patients at risk of PN, pharmacists can utilize the mnemonic MEDIC as shown in Table 2. | [1,21–34] |
| 4 | Common clinical symptoms for PN include tingling, numbness and nerve pain. Pharmacists can employ initial evaluation tools, such as clinical and lifestyle history, screening questionnaires (such as A Simple SCreening Tool (ACT), Douleur Neuropathique 4 (DN4), Neuropathic Pain Questionnaire (NPQ)) and sensory examinations (if local regulations permit), to assess patients with PN in the pharmacy setting. | [2,21,35–43] |
| 5 | Management of PN patients by pharmacists is recommended to follow the treatment algorithm in Figure 3. Pharmacists may recommend pharmacological approaches, including a complex of therapeutic doses of B vitamins (B1, B6, B12), low-dose pain relievers, and topical treatments for managing early PN. | [34,44–72] |
| 6 | Pharmacists should provide clear counselling to patients regarding the use of pharmacological options such as a complex of therapeutic doses of B vitamins (B1, B6, B12), including guidance on appropriate dosing, duration of therapy and potential side effects. This ensures patient safety while contributing to improved overall care. | [8–12,14,16,64,73,74] |


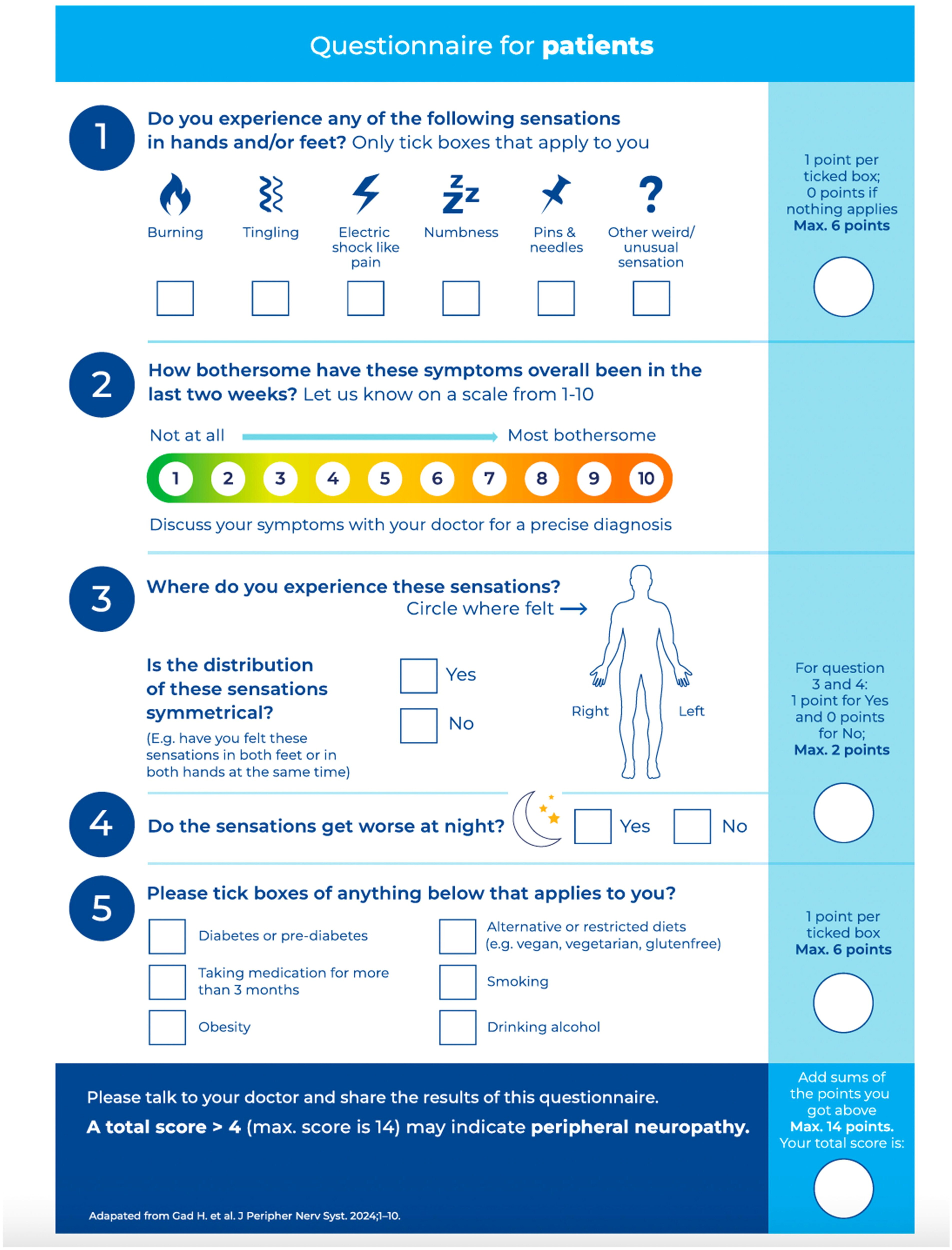


**Figure A1.** ACT patient questionnaire


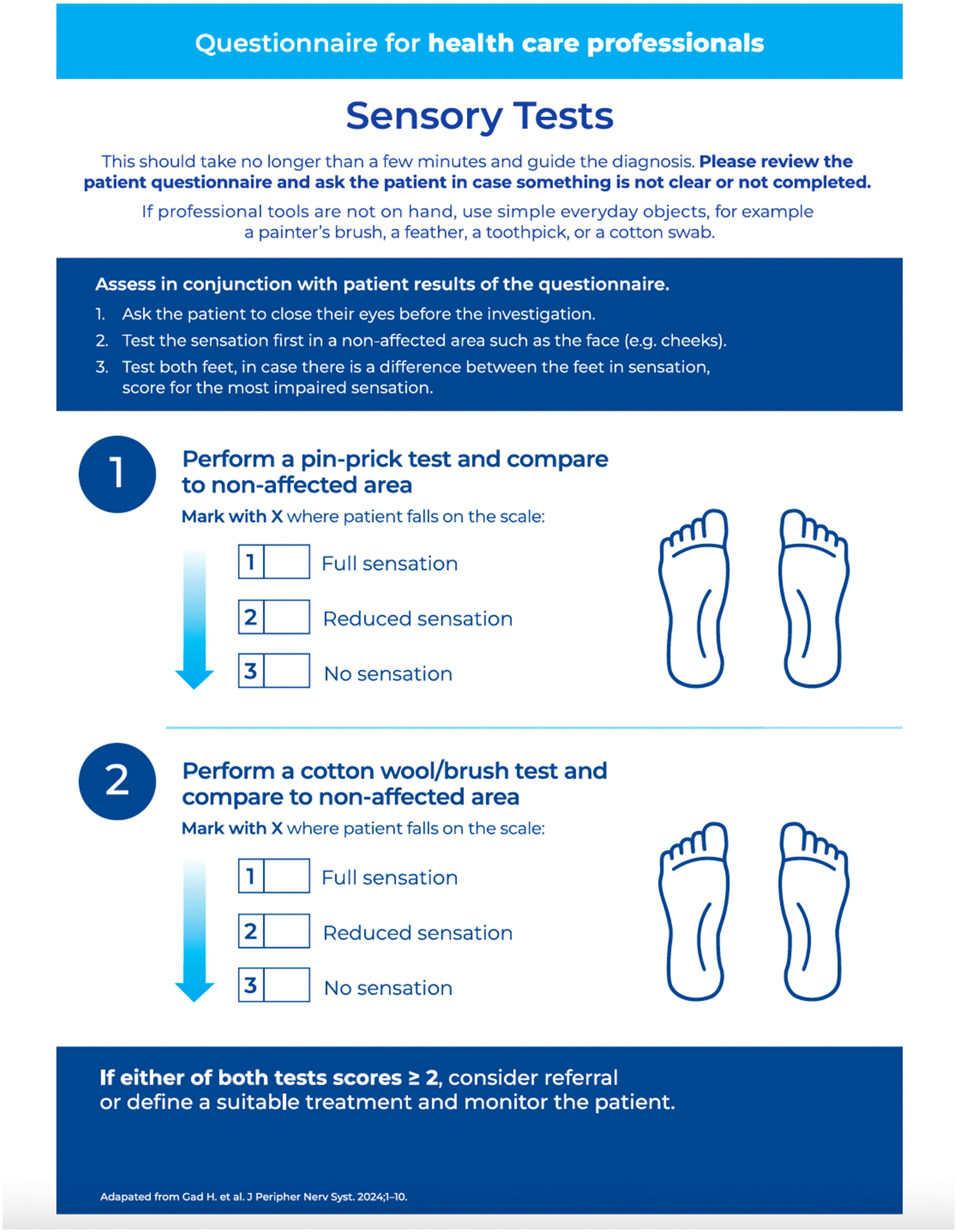


**Figure A2.** ACT healthcare professional questionnaire


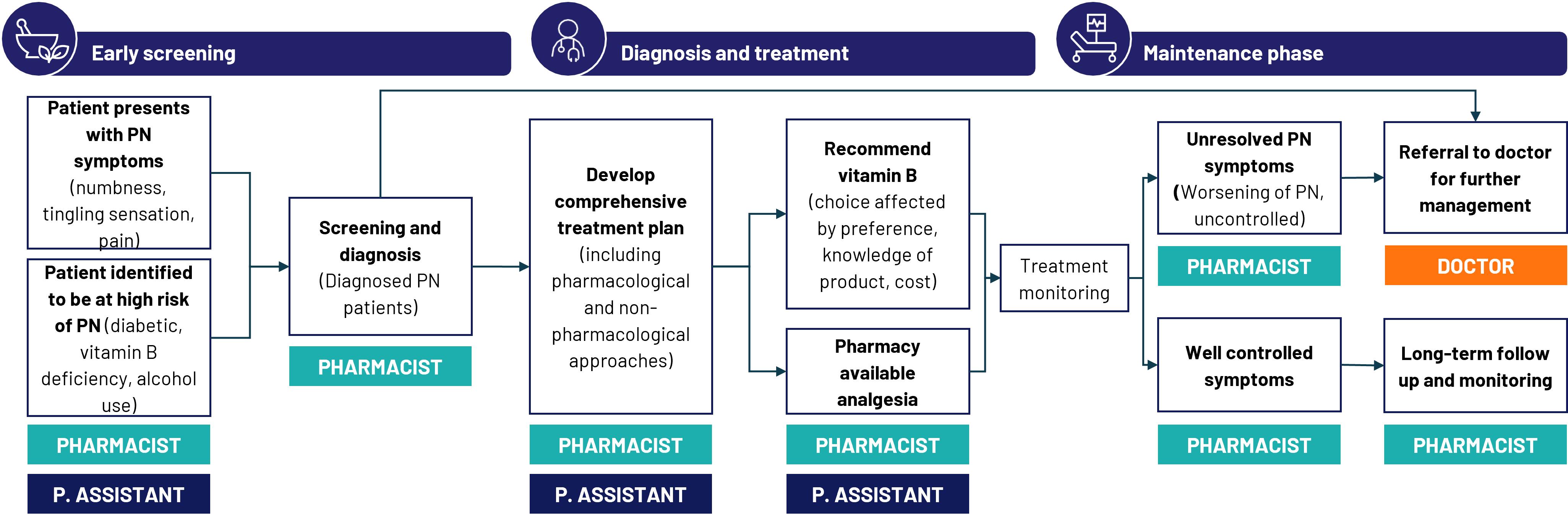


**Figure A3.** Treatment algorithm presented in Round 1 of the Delphi Survey

**References**

1. Malik, R.A.; Andag‐Silva, A.; Dejthevaporn, C.; Hakim, M.; Koh, J.S.; Pinzon, R.; Sukor, N.; Wong, K.S. Diagnosing Peripheral Neuropathy in South‐East Asia: A Focus on Diabetic Neuropathy. *J Diabetes Investig* **2020**, *11*, 1097–1103, doi:10.1111/jdi.13269.

2. Gad, H.; Kalra, S.; Pinzon, R.; Garcia, R.N.; Yotsombut, K.; Coetzee, A.; Nafach, J.; Lim, L.-L.; Fletcher, P.E.; Lim, V.; et al. Earlier Diagnosis of Peripheral Neuropathy in Primary Care: A Call to Action. *Journal of the Peripheral Nervous System* **2024**, *29*, 28–37, doi:10.1111/jns.12613.

3. Ponirakis, G.; Elhadd, T.; Chinnaiyan, S.; Hamza, A.H.; Sheik, S.; Kalathingal, M.A.; Anodiyil, M.S.; Dabbous, Z.; Siddique, M.A.; Almuhannadi, H.; et al. Prevalence and Risk Factors for Diabetic Neuropathy and Painful Diabetic Neuropathy in Primary and Secondary Healthcare in Qatar. *Journal of Diabetes Investigation* **2021**, *12*, 592–600, doi:10.1111/jdi.13388.

4. Ponirakis, G.; Elhadd, T.; Al Ozairi, E.; Brema, I.; Chinnaiyan, S.; Taghadom, E.; Al Kandari, J.; Al Wotayan, R.; Al Ozairi, A.; Aljohani, N.; et al. Prevalence and Risk Factors for Diabetic Peripheral Neuropathy, Neuropathic Pain and Foot Ulceration in the Arabian Gulf Region. *Journal of Diabetes Investigation* **2022**, *13*, 1551–1559, doi:10.1111/jdi.13815.

5. Srinivasan, A.V.; Paranjothi, S.; Bhattacharyya, K.B.; Maji, D.; Hazra, D.K.; Talwalkar, P.; Shah, S.; Sahay, R.K.; Khandelwal, D. Consensus Recommendations for the Management of Peripheral Neuropathy in India. *Journal of the Indian Medical Association* *116*, 45–55.

6. Ilardo, M.L.; Speciale, A. The Community Pharmacist: Perceived Barriers and Patient-Centered Care Communication. *International Journal of Environmental Research and Public Health* **2020**, *17*, 536, doi:10.3390/ijerph17020536.

7. Muharram, F.R.; Sulistya, H.A.; Swannjo, J.B.; Firmansyah, F.F.; Rizal, M.M.; Izza, A.; Isfandiari, M.A.; Ariningtyas, N.D.; Romdhoni, A.C. The Indonesia Health Workforce Quantity and Distribution 2024, 2024.03.31.24305126.

8. Mizranita, V.; Ponto, T.; Sipana, B. Overview of Indonesian Community Pharmacy: Understanding Practice Changes. *JPSCR: Journal of Pharmaceutical Science and Clinical Research* **2024**, *9*, 164–173, doi:10.20961/jpscr.v9i1.80498.

9. Buss, V.H.; Shield, A.; Kosari, S.; Naunton, M. The Impact of Clinical Services Provided by Community Pharmacies on the Australian Healthcare System: A Review of the Literature. *Journal of Pharmaceutical Policy and Practice* **2018**, *11*, 22, doi:10.1186/s40545-018-0149-7.

10. Yotsombut, K.; Pengsuparp, T.; Palapinyo, S. Community Pharmacy Practice in Thailand: The Diversity of Practice. *Research in Social and Administrative Pharmacy* **2012**, *8*, e9–e10, doi:10.1016/j.sapharm.2012.08.024.

11. Juperi, R.N.A.A.; Goh, H.P.; Rehman, I.U.; Lee, K.S.; Ming, L.C.; Hermansyah, A. Evaluating Healthcare Professionals’ Perceptions of Dispensing Separation and Sale of Pharmacy Medicine in Brunei Darussalam. *Journal of Pharmaceutical Policy and Practice* **2023**, *16*, 101, doi:10.1186/s40545-023-00594-5.

12. Vreeland, K.C.B.; Arteta, L.C.G.; Basto, K.M.L.; Cabaron, C.K.D.; Chicombing, R.J.C.; Dagayloan, A.J.B.; Lachica, B.J.D.; Purisima, Y.V.; Torres, J.R.B.; Santiago, C.D.; et al. Public Awareness, Expectations, and Views on Community Pharmacist’s Roles and Practices in the Philippines. *GSC Biological and Pharmaceutical Sciences* **2022**, *20*, 022–031, doi:10.30574/gscbps.2022.20.1.0260.

13. Siaw, M.Y.L.; Toh, J.H.; Lee, J.Y.-C. Patients’ Perceptions of Pharmacist-Managed Diabetes Services in the Ambulatory Care and Community Settings within Singapore. *Int J Clin Pharm* **2018**, *40*, 403–411, doi:10.1007/s11096-018-0591-2.

14. Hassali, M.A.A. Role of Community Pharmacists in Medication Management: Current and Future Prospects in Asia. **2017**.

15. Zeldin, E.R.; Goddard, A.R.; Boyle, M.S.; Madathil, R.L.; Rosenvall, E.; Majithia, K.A.; Morrison, E.J. An Overview of the Non‐procedural Treatment Options for Peripheral Neuropathic Pain. *Muscle Nerve* **2025**, *71*, 791–801, doi:10.1002/mus.28286.

16. Calderon-Ospina, C.-A.; Nava-Mesa, M.O.; Paez-Hurtado, A.M. Update on Safety Profiles of Vitamins B1, B6, and B12: A Narrative Review. *Ther Clin Risk Manag* **2020**, *16*, 1275–1288, doi:10.2147/TCRM.S274122.

17. Meyer, H.E.; Willett, W.C.; Fung, T.T.; Holvik, K.; Feskanich, D. Association of High Intakes of Vitamins B_6_ and B_12_ From Food and Supplements With Risk of Hip Fracture Among Postmenopausal Women in the Nurses’ Health Study. *JAMA Netw Open* **2019**, *2*, e193591, doi:10.1001/jamanetworkopen.2019.3591.

18. Primary Care Pathway: Suspected Diabetic Peripheral Neuropathy.Pdf.

19. Haanpää, M.L.; Gourlay, G.K.; Kent, J.L.; Miaskowski, C.; Raja, S.N.; Schmader, K.E.; Wells, C.D. Treatment Considerations for Patients With Neuropathic Pain and Other Medical Comorbidities. *Mayo Clin Proc* **2010**, *85*, S15–S25, doi:10.4065/mcp.2009.0645.

20. American Society of Health-System Pharmacists ASHP Statement on the Pharmacist’s Role in Clinical Pharmacokinetic Monitoring. **1998**.

21. Castelli, G.; Desai, K.M.; Cantone, R.E. Peripheral Neuropathy: Evaluation and Differential Diagnosis. *Am Fam Physician* **2020**, *102*, 732–739.

22. Cavaye, D.; Lehnbom, E.C.; Laba, T.-L.; El-Boustani, E.; Joshi, R.; Webster, R. Considering Pharmacy Workflow in the Context of Australian Community Pharmacy: A Pilot Time and Motion Study. *Research in Social and Administrative Pharmacy* **2018**, *14*, 1157–1162, doi:10.1016/j.sapharm.2018.01.003.

23. Mafukidze, A.T.; Calnan, M.; Furin, J. Peripheral Neuropathy in Persons with Tuberculosis. *J Clin Tuberc Other Mycobact Dis* **2016**, *2*, 5–11, doi:10.1016/j.jctube.2015.11.002.

24. Yang, R.; Yu, H.; Wu, J.; Chen, H.; Wang, M.; Wang, S.; Qin, X.; Wu, T.; Wu, Y.; Hu, Y. Metformin Treatment and Risk of Diabetic Peripheral Neuropathy in Patients with Type 2 Diabetes Mellitus in Beijing, China. *Front Endocrinol (Lausanne)* **2023**, *14*, 1082720, doi:10.3389/fendo.2023.1082720.

25. Alharbi, T.J.; Tourkmani, A.M.; Abdelhay, O.; Alkhashan, H.I.; Al-Asmari, A.K.; Rsheed, A.M.B.; Abuhaimed, S.N.; Mohammed, N.; AlRasheed, A.N.; AlHarbi, N.G. The Association of Metformin Use with Vitamin B12 Deficiency and Peripheral Neuropathy in Saudi Individuals with Type 2 Diabetes Mellitus. *PLOS ONE* **2018**, *13*, e0204420, doi:10.1371/journal.pone.0204420.

26. Hicks, C.W.; Wang, D.; Windham, B.G.; Matsushita, K.; Selvin, E. Prevalence of Peripheral Neuropathy Defined by Monofilament Insensitivity in Middle-Aged and Older Adults in Two US Cohorts. *Sci Rep* **2021**, *11*, 19159, doi:10.1038/s41598-021-98565-w.

27. Head, K.A. Peripheral Neuropathy: Pathogenic Mechanisms and Alternative Therapies. *Alternative Medicine Review* **2006**, *11*, 294–329.

28. Liu, X.; Xu, Y.; An, M.; Zeng, Q. The Risk Factors for Diabetic Peripheral Neuropathy: A Meta-Analysis. *PLOS ONE* **2019**, *14*, e0212574, doi:10.1371/journal.pone.0212574.

29. Verma, S.; Simpson, D.M. Peripheral Neuropathy in HIV Infection. In *Handbook of Clinical Neurology*; HIV/AIDS and the Nervous System; Elsevier, 2007; Vol. 85, pp. 129–137.

30. Sithinamsuwan, P.; Punthanamongkol, S.; Valcour, V.; Onsanit, S.; Nidhinandana, S.; Thitivichianlert, S.; Shikuma, C. Frequency and Characteristics of HIV-Associated Sensory Neuropathy Among HIV Patients in Bangkok, Thailand. *J Acquir Immune Defic Syndr* **2008**, *49*, 456–458, doi:10.1097/QAI.0b013e318186eb03.

31. Arnold, R.; Issar, T.; Krishnan, A.V.; Pussell, B.A. Neurological Complications in Chronic Kidney Disease. *JRSM Cardiovascular Disease* **2016**, *5*, 2048004016677687, doi:10.1177/2048004016677687.

32. Cai, X.-Y.; Li, W.-L.; Ge, S.-W.; Xu, G. Peripheral Neuropathy Associated with Higher Mortality in Population with Chronic Kidney Disease: National Health and Nutrition Examination Surveys. *Kidney Dis (Basel)* **2024**, *10*, 79–88, doi:10.1159/000535481.

33. Gondhali, G.; Kundalwal, A.; Takalkar, A.A. Peripheral Neuropathy in Chronic Kidney Diseases: Prevalence and Its Correlates. *Int J Adv Med* **2019**, *7*, 22, doi:10.18203/2349-3933.ijam20195621.

34. Pinzon, R.; Schellack, N.; Matawaran, B.J.; Tsang, M.W.; Deerochanawong, C.; Hiew, F.L.; Nafach, J.; Khadilkar, S. Clinical Recommendations for the Use of Neurotropic B Vitamins (B1, B6, and B12) for the Management of Peripheral Neuropathy: Consensus from a Multidisciplinary Expert Panel. *The Journal of the Association of Physicians of India* **2023**, *71*, 11–12, doi:10.59556/japi.71.0290.

35. Gad, H.; Dinamarca, J.L.; Fletcher, P.; Chen Ku, C.H.; Lira, R.; Longa, J.; Mendivil, C.; Palacios, L.; Pedrosa, H.; Román Pintos, L.M.; et al. Earlier Diagnosis of Peripheral Neuropathy in Primary Care in Latin America Using a Simple Screening Tool (ACT). *Current Medical Research and Opinion* **2025**, *41*, 93–104, doi:10.1080/03007995.2024.2443109.

36. Purwata, T.E.; Sadeli, H.A.; Anwar, Y.; Amir, D.; Asnawi, C.; Rahmawati, D.; Partoatmodjo, L.; Aulina, S.; Widyadarma, P.E.; Dalhar, M.; et al. Characteristics of Neuropathic Pain in Indonesia: A Hospital Based National Clinical Survey. *Neurology Asia* **2015**, *20*, 389–394.

37. Ching, S.M.; Lee, K.W.; Khan, A.H.K.Y.; Devaraj, N.K.; Cheong, A.T.; Yap, S.F.; Hoo, F.K.; Sulaiman, W.A.W.; Loh, W.C.; Chong, S.H.; et al. Prevalence and Factors Associated with Peripheral Neuropathy in a Setting of Retail Pharmacies in Malaysia–A Cross-Sectional Study. *PLOS ONE* **2024**, *19*, e0307093, doi:10.1371/journal.pone.0307093.

38. Mense, S.; Gerwin, R.D. *Muscle Pain: Understanding the Mechanisms*; Springer Science & Business Media, 2010; ISBN 978-3-540-85021-2.

39. DN4 Questionnaire.

40. Krause, S.J.; Backonja, M.-M. Development of a Neuropathic Pain Questionnaire. *The Clinical Journal of Pain* **2003**, *19*, 306.

41. Yang, Z.; Zhang, Y.; Chen, R.; Huang, Y.; Ji, L.; Sun, F.; Hong, T.; Zhan, S. Simple Tests to Screen for Diabetic Peripheral Neuropathy. *Cochrane Database Syst Rev* **2018**, *2018*, CD010975, doi:10.1002/14651858.CD010975.pub2.

42. Blackwell, W.; Khan, D. Penicillin Allergy Testing by Allergy Trained Pharmacists in Hospitalized Patients. *Journal of Allergy and Clinical Immunology* **2020**, *145*, AB161, doi:10.1016/j.jaci.2019.12.423.

43. The Medical Council of Thailand *The Medical Profession Act B.E. 2525 (1982)*; The Medical Council: Bangkok, 1996; ISBN 978-974-89802-4-9.

44. Hakim, M.; Kurniani, N.; Pinzon, R.T.; Tugasworo, D.; Basuki, M.; Haddani, H.; Pambudi, P.; Fithrie, A.; Wuysang, A.D. Management of Peripheral Neuropathy Symptoms with a Fixed Dose Combination of High-Dose Vitamin B1, B6 and B12: A 12-Week Prospective Non-Interventional Study in Indonesia. *Asian J Med Sci* **2018**, *9*, 32–40, doi:10.3126/ajms.v9i1.18510.

45. National Health and Medical Research Council Nutrient Reference Values for Australia and New Zealand - Thiamin Available online: https://www.eatforhealth.gov.au/nutrient-reference-values/nutrients/thiamin (accessed on 19 September 2025).

46. Southeast Asia Public Health Nutrition Network; Tee, E.S.; Nutrition Society of Malaysia; Florentino, R.F.; Southeast Asia Public Health Nutrition Network; Nutrition Foundation of the Philippines, Inc; Chongviriyaphan, N.; Southeast Asia Public Health Nutrition Network; Nutrition Association of Thailand; Ridwan, H.; et al. Review of Recommended Energy and Nutrient Intake Values in Southeast Asian Countries. *Mal J Nutr* **2023**, *29*, doi:10.31246/mjn-2023-29-2-rni-rda-sea-review.

47. Recommended Dietary Allowances Available online: https://www.healthhub.sg/well-being-and-lifestyle/food-diet-and-nutrition/recommended_dietary_allowances (accessed on 19 September 2025).

48. Woelk, H.; Lehrl, S.; Bitsch, R.; Kopcke, W. BENFOTIAMINE IN TREATMENT OF ALCOHOLIC POLYNEUROPATHY: AN 8-WEEK RANDOMIZED CONTROLLED STUDY (BAP I STUDY). *Alcohol and Alcoholism* **1998**, *33*, 631–638, doi:10.1093/alcalc/33.6.631.

49. Haupt, E.; Ledermann, H.; Köpcke, W. Benfotiamine in the Treatment of Diabetic Polyneuropathy a Three-Week Randomized, Controlled Pilot Study (BEDIP Study). *CP* **2005**, *43*, 71–77, doi:10.5414/CPP43071.

50. Stracke, H.; Gaus, W.; Achenbach, U.; Federlin, K.; Bretzel, R.G. Benfotiamine in Diabetic Polyneuropathy (BENDIP): Results of a Randomised, Double Blind, Placebo-Controlled Clinical Study. *Exp Clin Endocrinol Diabetes* **2008**, *116*, 600–605, doi:10.1055/s-2008-1065351.

51. Norio, O.; Tsuneo, F.; Akito, H. Functional Limitations in Thiamine Deficiency Neuropathy: FIM Score Improvement With Treatment. *Journal of Clinical Neuromuscular Disease* **2006**, *7*, 104–109, doi:10.1097/01.cnd.0000203643.84628.a1.

52. Lei, Y.; Zheng, M.-H.; Huang, W.; Zhang, J.; Lu, Y. Wet Beriberi with Multiple Organ Failure Remarkably Reversed by Thiamine Administration. *Medicine (Baltimore)* **2018**, *97*, e0010, doi:10.1097/MD.0000000000010010.

53. Di Marco, S.; Pilati, L.; Brighina, F.; Fierro, B.; Cosentino, G. Wernicke-Korsakoff Syndrome Complicated by Subacute Beriberi Neuropathy in an Alcoholic Patient. *Clinical Neurology and Neurosurgery* **2018**, *164*, 1–4, doi:10.1016/j.clineuro.2017.11.006.

54. National Health and Medical Research Council Nutrient Reference Values for Australia and New Zealand - Nutrients Available online: https://www.eatforhealth.gov.au/nutrient-reference-values/nutrients (accessed on 19 September 2025).

55. Moriwaki, K.; Kanno, Y.; Nakamoto, H.; Okada, H.; Suzuki, H. Vitamin B6 Deficiency in Elderly Patients on Chronic Peritoneal Dialysis. *Adv Perit Dial* **2000**, *16*, 308–312.

56. Ellis, J.M.; Folkers, K.; Levy, M.; Shizukuishi, S.; Lewandowski, J.; Nishii, S.; Schubert, H.A.; Ulrich, R. Response of Vitamin B-6 Deficiency and the Carpal Tunnel Syndrome to Pyridoxine. *Proc Natl Acad Sci U S A* **1982**, *79*, 7494–7498, doi:10.1073/pnas.79.23.7494.

57. Okada, H.; Moriwaki, K.; Kanno, Y.; Sugahara, S.; Nakamoto, H.; Yoshizawa, M.; Suzuki, H. Vitamin B6 Supplementation Can Improve Peripheral Polyneuropathy in Patients with Chronic Renal Failure on High‐flux Haemodialysis and Human Recombinant Erythropoietin. *Nephrology Dialysis Transplantation* **2000**, *15*, 1410–1413, doi:10.1093/ndt/15.9.1410.

58. Levin, E.R.; Hanscom, T.A.; Fisher, M.; Lauvstad, W.A.; Lui, A.; Ryan, A.; Glockner, D.; Levin, S.R. The Influence of Pyridoxine in Diabetic Peripheral Neuropathy. *Diabetes Care* **1981**, *4*, 606–609, doi:10.2337/diacare.4.6.606.

59. Janka, H.U.; Rietzel, S.; Mehnert, H. Der Einfluß von Neurobion auf die Temperatursensibilität bei Patienten mit diabetischer Polyneuropathie. In *Pharmakologie und klinische Anwendung hochdosierter B-Vitamine*; Rietbrock, N., Ed.; Steinkopff: Heidelberg, 1991; pp. 87–97 ISBN 978-3-642-85411-8.

60. National Health and Medical Research Council Nutrient Reference Values for Australia and New Zealand - Vitamin B12 Available online: https://www.eatforhealth.gov.au/nutrient-reference-values/nutrients/vitamin-b12 (accessed on 19 September 2025).

61. Didangelos, T.; Karlafti, E.; Kotzakioulafi, E.; Margariti, E.; Giannoulaki, P.; Batanis, G.; Tesfaye, S.; Kantartzis, K. Vitamin B12 Supplementation in Diabetic Neuropathy: A 1-Year, Randomized, Double-Blind, Placebo-Controlled Trial. *Nutrients* **2021**, *13*, 395, doi:10.3390/nu13020395.

62. Roessler, F.C.; Wolff, S. Rapid Healing of a Patient with Dramatic Subacute Combined Degeneration of Spinal Cord: A Case Report. *BMC Res Notes* **2017**, *10*, 18, doi:10.1186/s13104-016-2344-4.

63. Castelli, M.C.; Friedman, K.; Sherry, J.; Brazzillo, K.; Genoble, L.; Bhargava, P.; Riley, M.G.I. Comparing the Efficacy and Tolerability of a New Daily Oral Vitamin B12 Formulation and Intermittent Intramuscular Vitamin B12 in Normalizing Low Cobalamin Levels: A Randomized, Open-Label, Parallel-Group Study. *Clinical Therapeutics* **2011**, *33*, 358-371.e2, doi:10.1016/j.clinthera.2011.03.003.

64. Schellack, N.; Yotsombut, K.; Sabet, A.; Nafach, J.; Hiew, F.L.; Kulkantrakorn, K. Expert Consensus on Vitamin B6 Therapeutic Use for Patients: Guidance on Safe Dosage, Duration and Clinical Management. *DHPS* **2025**, *17*, 97–108, doi:10.2147/DHPS.S499941.

65. Blaibel, D.; Fernandez, C.J.; Pappachan, J.M. Non-Pharmacological Interventions for Diabetic Peripheral Neuropathy: Are We Winning the Battle? *World J Diabetes* **2024**, *15*, 579–585, doi:10.4239/wjd.v15.i4.579.

66. Martel, J.L.; Doshi, H.; Sina, R.E.; Franklin, D.S. Vitamin B1 (Thiamine). In *StatPearls*; StatPearls Publishing: Treasure Island (FL), 2025.

67. Vitamin B12 - Health Professional Fact Sheet Available online: https://ods.od.nih.gov/factsheets/VitaminB12-HealthProfessional/ (accessed on 21 July 2025).

68. Yoshioka, K.; Tanaka, K. Effect of Methylcobalamin on Diabetic Autonomic Neuropathy as Assessed by Power Spectral Analysis of Heart Rate Variations. *Horm Metab Res* **1995**, *27*, 43–44, doi:10.1055/s-2007-979907.

69. Taniguchi, H.; Ejiri, K.; Baba, S. Improvement of Autonomic Neuropathy after Mecobalamin Treatment in Uremic Patients on Hemodialysis. *Clin Ther* **1987**, *9*, 607–614.

70. Ekabe, C.J.; Kehbila, J.; Abanda, M.H.; Kadia, B.M.; Sama, C.-B.; Monekosso, G.L. Vitamin B12 Deficiency Neuropathy; a Rare Diagnosis in Young Adults: A Case Report. *BMC Res Notes* **2017**, *10*, 72, doi:10.1186/s13104-017-2393-3.

71. Solomon, L.R. Diabetes as a Cause of Clinically Significant Functional Cobalamin Deficiency. *Diabetes Care* **2011**, *34*, 1077–1080, doi:10.2337/dc11-0009.

72. Solomon, L.R. Vitamin B_12_ -Responsive Neuropathies: A Case Series. *Nutritional Neuroscience* **2016**, *19*, 162–168, doi:10.1179/1476830515Y.0000000006.

73. Singapore Pharmacy Council Community Pharmacist Available online: https://www.spc.gov.sg/for-professionals/types-of-pharmacists/community-pharmacist/ (accessed on 18 July 2025).

74. Inoue, Y.; Takikawa, M.; Morita, Y.; Takao, K.; Kanamoto, I.; Sugibayashi, K. A Comparison of Pharmacists’ Role Functions across Various Nations: The Importance of Screening. *Research in Social and Administrative Pharmacy* **2016**, *12*, 347–354, doi:10.1016/j.sapharm.2015.05.005.
